# Multiple-element exposure in early pregnancy and birth defects: a nested case–control study based on the China birth cohort study

**DOI:** 10.3389/fnut.2026.1722672

**Published:** 2026-03-09

**Authors:** Weichunbai Zhang, Jianming Zhang, Jing Du, Qinglin Ma, Tianhao Shan, Shanshan Sun, Yunjia Yang, Hong Li, Gang Li, Yan Song, Jilong Yao, Yi Yang

**Affiliations:** 1 NHC Key Laboratory of Food Safety Risk Assessment, China National Center for Food Safety Risk Assessment, Beijing, China; 2 Shenzhen Maternity and Child Healthcare Hospital, Southern Medical University, Shenzhen, China; 3 Beijing Key Laboratory of Diagnostic and Traceability Technologies for Food Poisoning, Beijing Center for Disease Prevention and Control, Beijing, China

**Keywords:** birth defect, China birth cohort study, early pregnancy, multiple-element exposure, nested case–control study

## Abstract

**Background:**

Birth defects have become the leading cause of death among children, placing a heavy burden on society and families. Various elements from the environment may be associated with birth defects in the fetus, but few prospective studies have investigated the relationship between multiple-element exposure in the first trimester and birth defects.

**Methods:**

In this study, 271 cases were matched to 542 controls for age (±2 years) and gestational age (±2 weeks). We measured the concentrations of 17 elements in urine samples collected from pregnant women during the first trimester and used conditional logistic regression models to estimate odds ratios (ORs) and 95% confidence intervals (95% CIs). The BKMR model was used to evaluate the correlation between element mixed exposure and birth defects.

**Results:**

In the single-metal multivariate model, aluminum (Al), chromium (Cr), manganese (Mn), iron (Fe), nickel (Ni), and zinc (Zn) were negatively associated with total birth defects. Restricted cubic splines revealed linear or nonlinear dose-responsive relationships between either Ni or Zn and the risk of birth defects. BKMR results showed that birth defects showed a trend of first slightly rising and then declining; when the element mixture was above the 50th percentile, it was significantly negatively associated with total birth defects. Ni and Zn showed an obvious trend of slight increase and then rapid decrease, indicating that there was a dose–response relationship between these elements and birth defects. There were interactions between Ni and the other five elements.

**Conclusion:**

The mixture of high levels of Al, Cr, Mn, Fe, Ni, and Zn in urine during the first trimester of pregnancy was related to a reduction in total birth defects. In particular, the influence of Ni and Zn on total birth defects should be considered.

## Introduction

Generally, abnormal structural or functional metabolism during embryonic or fetal development is considered a birth defect (1). According to the World Health Organization (WHO), the common types of birth defects include congenital heart disease (CHD), neural tube malformation, Down syndrome, and congenital limb abnormalities WHO (2). Birth defects are the main causes of fetal death, early abortion, perinatal death and infant death. Among surviving children, most birth defects cause disability, which seriously affects quality of life, places serious disease and economic burdens on families and society and is a global public health problem (3, 4). Approximately 3% to 4% of infants aged 0 to 1 have serious birth defects, and birth defects have become the leading cause of infant death in some countries and the fifth leading cause of shortened life expectancy (5). According to WHO estimates, approximately 10% of neonatal deaths occur every year, which is significantly higher than that in 2004 (7%) (6, 7). The current incidence rate of birth defects in China is approximately 2.5%, and the three leading birth defects are congenital heart defects, urinary system and genital organ malformations, and chromosomal abnormalities (8). It was estimated that 250,000 newborns would suffer from birth defects in approximately 10 million newborns every year in China, according to the China Birth Cohort Study (CBCS) results (8).

Environmental and genetic factors are the most common causes of birth defects (9, 10). A meta-analysis of congenital heart defects suggested that metals as well as smoking, vehicle exhaust components, pesticides, organic solvents and proximity to landfills were associated with the incidence of CHDs (11). Among all environmental factors, element exposure is one of the most important because element exposure could have multiple effects on birth defects via multiple exposure pathways. On the one hand, some elements, such as iron (Fe) and zinc (Zn), are essential elements for human health. Several previous studies have suggested that Zn was negatively correlated with various birth defects, such as neural tube defects (NTDs), gastrointestinal malformations and cleft lip and palate (12–14). Several studies also indicated that the intake of high-level Fe could significantly reduce the risk of diaphragmatic hernia and CHDs in offspring (15, 16). A systematic review showed that there was insufficient evidence for the protective effects of elements (Zn and Fe) against fetal CHDs (17). On the other hand, toxic metals, as the main environmental pollutants, such as lead (Pb) and cadmium (Cd), are also harmful to human health. Some previous studies showed that high levels of blood Pb in pregnant women were associated with increased risks of NTDs (18, 19). A meta-analysis study suggested that exposure to arsenic (As) and Pb was significantly associated with an increased risk of CHDs (20). In recent years, there has been increasing evidence that exposure to inorganic elements is associated with the risk of birth defects. However, most epidemiological studies focus on the effect of single-element exposure on birth defects, ignoring the effect of coexposure to multiple elements and the importance of the degree of exposure (21, 22).

As common components of and pollutants in the natural environment, a variety of elements coexist in the ecosystem and enter the body through air, drinking water, food, and medicines. Therefore, pregnant women can be exposed to all kinds of elements, including essential elements and toxic elements, by multiple pathways. To date, most of the previous studies on inorganic elements and birth defects have been retrospective (23). For the particularity of the nutritional and physiological status of pregnant women, the accuracy of retrospective studies was not good enough, which resulted in inconsistencies in several studies. In addition, previous studies have mainly focused on several single birth defects, such as coronary heart disease and neural tube defects (24–26). In fact, approximately 20%–30% of cases of birth defects are multiple congenital anomalies (MCAs) with more than one organ abnormality clinically. It was suggested that the commonality of environmental influences on birth defects should be considered (27–30). Therefore, this study employed a prospective nested case–control design, systematically collecting bio-specimens during early pregnancy to quantify co-exposure levels of multiple elements. By focusing on multi-element exposure scenarios that more closely reflect the complexly real exposure patterns, this approach addressed the limitations predominantly restricted to single-element analyses. It was expected that this study could provide new evidence for understanding the etiology of birth defects and lay a scientific foundation for establishing primary prevention strategies based on multi-element exposure assessment.

## Methods

### Study population

This population-based prospective nested case–control study was based on the Shenzhen subcenter of the CBCS. The CBCS was a prospective longitudinal, large cohort study and the first nationally based birth cohort study to establish birth cohorts covering a nationally representative geographic area to investigate risk factors for birth defects and formulate a reduction strategy (8). In this study, a total of 20,170 subjects were from the Shenzhen population in the CBCS from 2018 to 2021. All participants had urine samples in the first trimester (6 − 13 + 6 weeks of gestation) and received questionnaires so that relevant data could be collected (8). The questionnaire data mainly included age, date of the last menstrual period, educational level, household income, lifestyle habits, height and weight before pregnancy, birth defects of pregnant women and their spouses, conception methods, first pregnancy, prepregnancy disease status, and folic acid use.

**Figure 1:**
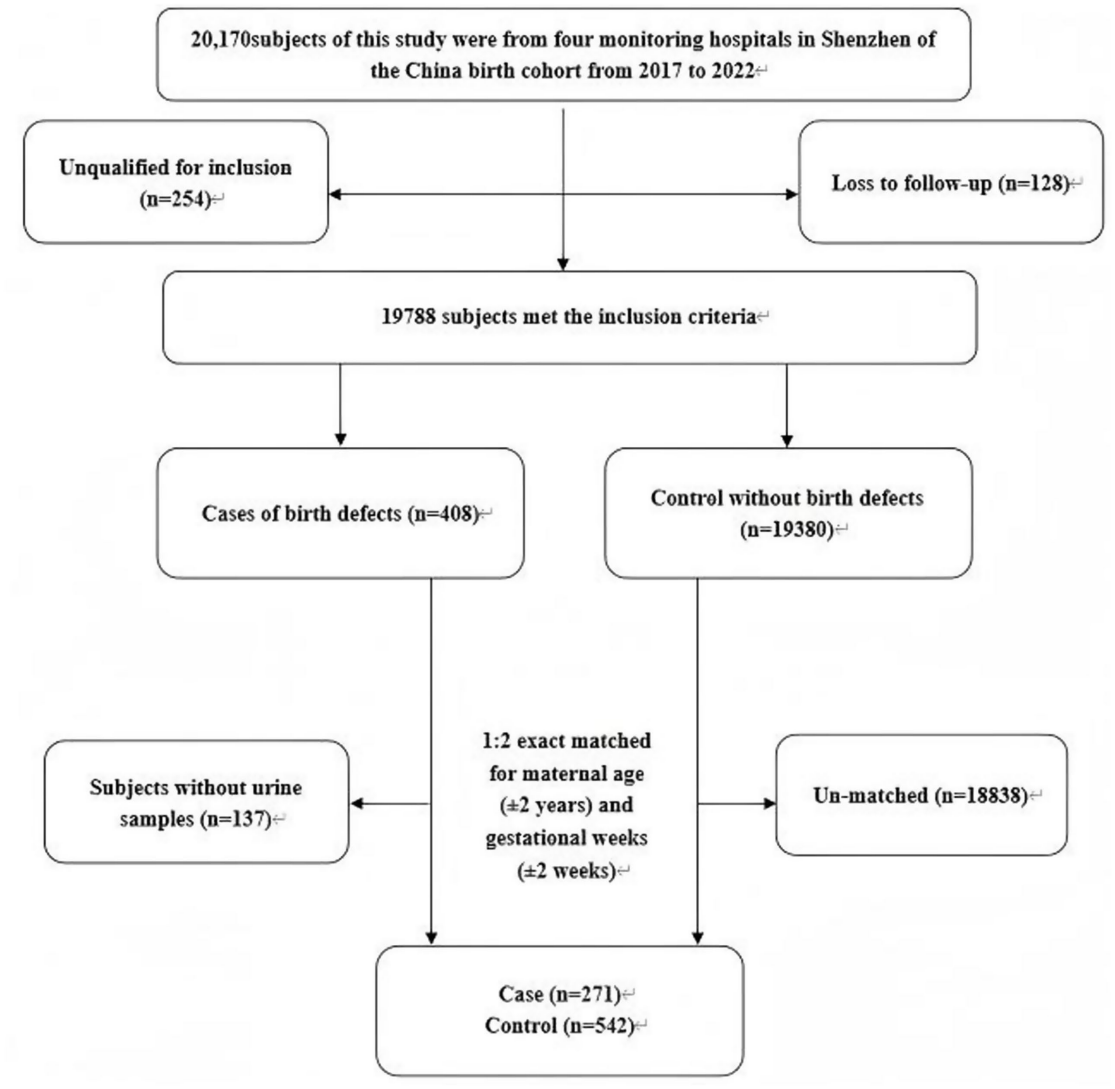
Flow chart of the study population.

Among the 20,170 subjects, 254 participants did not meet the inclusion criteria, and 128 participants could not be followed up. During this period, 408 cases of birth defects were found, which were detected from the time of investigation to the time of birth. In this study, birth defects are defined as structural, functional, or metabolic abnormalities that occur before the birth of an infant. All cases of birth defects were determined by experienced obstetricians, sonographers, and pediatricians after joint diagnosis in strict accordance with the International Classification of Diseases (ICD-10). For the subjects in the case group, after the exclusion of pregnant women without urine samples (*n* = 137), we included 271 cases in the birth defect group. In detail, there were 49 cases of craniofacial abnormalities, 44 cases of limb abnormalities, 61 cases of congenital heart disease, 53 cases of chromosomal abnormalities, 41 cases of urogenital system abnormalities and 23 cases of other abnormalities. The control group was randomly selected from live births and nonteratogenic infants in the cohort and matched 1:2 according to maternal age (±2 years) and gestational age at survey (±2 weeks) (Figure 1). The study complied with the Declaration of Helsinki and was approved by the Shenzhen Maternal and Child Health Hospital Ethics Committee.

### Urine sample preparation and elemental analysis

Fresh spot urine samples were collected from pregnant women in the first trimester in polypropylene bottles washed with 5% nitric acid and stored in a –80 °C freezer. Urine samples were thawed at room temperature during measurement, and after mixing, 200 μL of the urine sample was obtained and diluted 1:10 with a solution containing 0.05% tetramethylammonium hydroxide, 4% isopropanol and 0.01% ethylenediaminetetraacetic acid. Urine samples were analyzed by a NexION 2000 inductively coupled plasma mass spectrometer (PerkinElmer). Magnesium (Mg), aluminum (Al), calcium (Ca), chromium (Cr), manganese (Mn), Fe, nickel (Ni), copper (Cu), Zn, As, selenium (Se), strontium (Sr), Cd, iodine (I), barium (Ba), mercury (Hg), and Pb in the urine of pregnant women were analyzed in this study. Bismuth (Bi), scandium (Sc), germanium (Ge), rhodium (Rh), and indium (In) were mixed with the sample to be tested by online addition before being introduced into the atomizer as the internal standard solution. The test results were displayed in ng/mL. The limit of detection (LOD) of 17 elements ranged from 0.005 ng/mL to 0.458 ng/mL, as shown in Supplementary Table S1. Among them, Mg (0.5%), Al (6.5%), Cr (1.7%), Mn (0.4%), Ni (2.0%), Cd (0.1%), I (0.1%), and Hg (21.4%) have cases below the LOD. The concentrations of elements below the LOD were set to 1/2LOD. Urine creatinine concentrations were measured using a Hitachi Autoanalyzer 7,600 (Tokyo, Japan). All laboratory tests were performed at the Beijing Center for Disease Control and Prevention laboratory.

### Quality control

To ensure the accuracy and reliability of urine sample testing, we took the following measures during the urinalysis process. We evaluated the quality control samples every 20 samples to ensure the stability of the instrument. We chose Bi, Sc, Ge, Rh, and In as the internal standards to improve the detection accuracy. For the instrument analysis of the urine sample, the sample was injected blindly, and the operator who analyzed the urine elements was blinded to whether the sample belonged to the case group or the control group to avoid the influence of subjective factors on the analysis results. In addition, to avoid the influence of participants’ behaviors, such as drinking water, on the urine concentration, we also corrected the element concentration in the response samples by detecting the concentration of creatinine in the urine. For external quality assurance and control, the laboratory was accredited by the China National Accreditation Service for Conformity Assessment and mandatory approval of Chinese inspection agencies and laboratories and met all requirements. The laboratory had also been externally inspected by the National Center for Clinical Laboratories through proficiency testing.

### Covariates

The following variables obtained through the questionnaire were considered potential confounders: the basic information of pregnant women, lifestyle habits and pregnancy-related information. Basic information included maternal age, gestational age at the time of the survey, educational level, household income, and the height and weight of the pregnant woman. We assumed that the date of conception occurred on the first day of the last menstrual period and calculated gestational age based on the date and date of the survey. Educational level was divided into junior high school and below, high school, and university and above. Yearly average household income was divided into below 50,000 Chinese Yuan (CNY), 50,000–200,000 CNY and above 200,000 CNY. Prepregnancy body mass index (BMI) was calculated as weight (kg) divided by height (meters) squared. Lifestyle habits included mainly smoking and drinking. Smoking status was divided into never smoking and smoking (including former smoking and current smoking). Drinking status referred to whether or not you are currently drinking. Pregnancy-related information included family history of birth defects, conception method, first pregnancy, prepregnancy disease status, and folic acid use. Family history of birth defects referred to whether the baby’s parents and their parents and siblings had a birth defect disorder. Conception methods were divided into natural conception and other methods (test tube baby and artificial insemination, etc.). First pregnancy referred to whether the current pregnancy was the first time. Prepregnancy disease status referred to whether pregnant women suffered from diabetes, hypertension, periodontal disease, and reproductive tract inflammation in the first 3 months of pregnancy. Folic acid use referred to whether pregnant women took folic acid supplements.

### Statistical analysis

The basic characteristics of the case group and control group of birth defects were described, the mean ± standard deviation was used for continuous variables with normal distribution, and the *t*-test was used for comparison between groups. The median (interquartile range) was used for element concentration, and the comparison between groups was performed using the Mann–Whitney *U* test. Spearman’s rank correlation coefficient was used to evaluate the correlation between the urinary elemental concentrations. Odds ratios (ORs) and 95% confidence intervals (95% CIs) of urinary element concentrations and risk of birth defects were calculated by logistic regression. Model 1 was a simple logistic regression without adjustment for any confounders. Model 2 was a logistic regression adjusted for age, gestational age, educational level, household income, BMI, family history of birth defects, first pregnancy, conception method, prepregnancy disease status, and folic acid use.

For the statistically significant elements in Model 2, we conducted a series of sensitivity analyses to test the robustness of our estimates by excluding participants with a family history of birth defects, prepregnancy disease status or other conception methods and repeating the regression analysis. To overcome the inherent limitations of element-level analysis as a rank variable, a restricted cubic spline was used to analyze the elemental dose–response in a multielement model. Each elemental model used restricted cubic spline with nodes distributed at the 20th, 40th, 60th, and 80th percentiles of its distribution, and reference values (OR = 1) were set at the 10th percentile.

To estimate the joint effect and regress exposure–response relationships of multiple element exposure, Bayesian kernel machine regression (BKMR) was used to evaluate the correlation between multiple element concentrations and birth defects (31–34). Elements that were significantly associated with birth defects in the single element model were included in BKMR. BKMR models were fit using the Markov Chain Monte Carlo algorithm with 25,000 iterations using the Gaussian kernel. By comparing the estimated effect of all exposure factors at a specific percentile with the estimated effect of all exposure factors at their 50th percentile, we can fit the overall impact of mixed element exposure on birth defects. The exposure-response function was used to explore the relationship between a single element and birth defects while keeping the concentration of other elements at the median. Finally, the interaction between the effects of two metals on birth defects was studied, and the influence of one element at different quantile concentrations on the correlation between another element and birth defects was discussed by establishing a bivariate pairwise exposure–response function (the remaining elements were fixed at the median). The model calculated the conditional posterior inclusion probability (condPIP), which represented the probability of elements being included in the model. 0.5 was usually used as the threshold of condPIP to determine whether the exposure was important (35, 36).

All statistical analyses were performed using SPSS 21.0 and R 4.1.1. All statistical tests were two-sided, and *p* < 0.05 was considered statistically significant.

## Results

### Characteristics of the study population and urinary elements

This study included a total of 271 cases and 542 controls. The average age of the case group was 30.91 ± 4.17 years with the average gestational age of 10.30 ± 1.96 weeks, while the average age of the control group was 30.85 ± 4.04 years with the average gestational age of 10.30 ± 1.91 weeks. In the comparison of the two groups, it was found that the proportion of natural conception in the case group was lower (89.7%) than that in the control group (96.1%), and the proportion of prepregnancy disease in the case group was higher (25.5%) than that in the control group (17.9%) (*p* < 0.005). The other variables were not significantly different (Table 1).

**Table 1:** Basic characteristics of the study participants.

Characteristics	Case (*n* = 271)	Control (*n* = 542)	*p*-value^a^
Age (years)	30.91 ± 4.17	30.85 ± 4.04	0.832
Gestational age (weeks)	10.30 ± 1.96	10.30 ± 1.91	0.980
Educational level, *n* (%)			0.703
Junior high school and below	11 (4.0)	27 (5.0)	
High school	40 (14.8)	71 (13.1)	
University and above	220 (81.2)	444 (81.9)	
Household income, *n* (%)			0.229
Below 50,000 CNY^b^	13 (4.8)	14 (2.6)	
50,000–200,000 CNY^b^	96 (35.4)	188 (34.7)	
Above 200,000 CNY^b^	162 (59.8)	340 (62.7)	
Smoking status, *n* (%)			0.830
Smoking	4 (1.5)	7 (1.3)	
Never smoking	267 (98.5)	535 (98.7)	
Drinking status, *n* (%)			0.566
Drinking	7 (2.6)	18 (3.3)	
No drinking	264 (97.4)	524 (96.7)	
BMI(kg/m^2^)	21.32 ± 2.99	21.36 ± 3.06	0.863
First pregnancy, *n* (%)			0.190
Yes	102 (37.6)	230 (42.4)	
No	169 (62.4)	312 (57.6)	
Conception method, *n* (%)			<0.001
Natural conception	243 (89.7)	521 (96.1)	
Others	28 (10.3)	21 (3.9)	
Prepregnancy disease status^c^, *n* (%)			0.012
Yes	69 (25.5)	97 (17.9)	
No	202 (74.5)	445 (82.1)	
Family history of birth defects, *n* (%)			0.804
Yes	12 (4.4)	22 (4.1)	
No	259 (95.6)	520 (95.9)	
Folic acid use, *n* (%)			0.895
Using	225 (83.0)	448 (82.7)	
No using	46 (17.0)	94 (17.3)	

a *p*-values were derived from Student’s *t*-tests for continuous variables according to the data distribution and the chi-square test for the categorical variables.

b CNY was Chinese yuan.

c Prepregnancy disease including diabetes, hypertension, periodontal disease, and reproductive tract inflammation in the first 3 months of pregnancy.

In terms of element concentration in urine, the concentration of each element was normalized by creatinine. As shown in Table 2, the concentration of Al in the urine of the case group was 7.87 (4.10–18.010) μg/g, while that in the control group was 12.14 (5.08–30.99) μg/g (*p* < 0.001); the concentration of Fe in the urine of the case group was 19.13 (12.47–33.70) μg/g, while that in the control group was 25.97 (14.16–45.72) μg/g (*p* < 0.001). The concentrations of Cr, Ni, and Zn in the urine of the control group were also significantly higher than those in the urine of the case group (*p* < 0.05). Therefore, it was documented that the urine levels of Al, Cr, Fe, Ni, and Zn in the healthy control group were significantly higher than those in the case group (*p* < 0.05), while other elements were not significantly different between the case group and the control group. In addition, most of the elements in urine had significant but moderate correlations (Spearman correlation coefficients ranged from −0.091 to 0.677) (Supplementary Figure S1).

**Table 2:** Concentrations of urinary elements corrected by creatinine among study participants.

Elements	Case (*n* = 271)	Control (*n* = 542)	*p*-value^a^
Mg (mg/g)	25.24 (13.45–41.67)	27.02 (13.42–44.16)	0.218
Al (μg/g)	7.87 (4.10–18.01)	12.14 (5.08–30.99)	<0.001
Ca (mg/g)	34.70 (15.75–61.29)	35.32 (17.43–63.25)	0.804
Cr (μg/g)	0.31 (0.18–0.48)	0.39 (0.19–0.62)	0.002
Mn (μg/g)	0.57 (0.37–0.99)	0.68 (0.39–1.15)	0.060
Fe (μg/g)	19.13 (12.47–33.70)	25.97 (14.16–45.72)	<0.001
Ni (μg/g)	1.50 (0.92–2.37)	1.74 (0.93–3.44)	0.002
Cu (μg/g)	9.87 (7.31–13.71)	10.34 (7.94–15.76)	0.068
Zn (μg/g)	280.55 (189.02–380.02)	297.59 (203.46–443.54)	0.018
As (μg/g)	24.94 (16.81–38.03)	26.79 (16.56–47.67)	0.211
Se (μg/g)	27.18 (20.57–34.49)	28.17 (22.15–34.80)	0.113
Sr (μg/g)	91.25 (46.68–140.91)	92.68 (44.22–164.52)	0.369
Cd (μg/g)	0.75 (0.45–1.13)	0.74 (0.45–1.14)	0.932
I (μg/g)	90.68 (61.78–146.68)	95.62 (64.65–160.12)	0.299
Ba (μg/g)	36.52 (17.01–64.69)	41.66 (15.34–93.17)	0.164
Hg (μg/g)	0.09 (0.05–0.16)	0.10 (0.04–0.23)	0.243
Pb (μg/g)	0.77 (0.46–1.16)	0.71 (0.45–1.12)	0.583

a Mann–Whitney *U* test.

### Urinary elements and risk of birth defects

The results of the association between urinary element exposure concentrations and birth defects are shown in Table 3. In unadjusted model (Model 1), Al, Cr, Mn, Fe, Ni, Zn, and Hg in urine were negatively associated with total birth defects (*p* < 0.05). In Model 2, after adjusting for age, gestational age, educational level, household income, BMI, smoking status, drinking status, family history of birth defects, first pregnancy, conception method, prepregnancy disease status, and folic acid use, the urine concentrations of Al, Cr, Mn, Fe, Ni, and Zn were negatively associated with total birth defects (*p* < 0.05). In detail, for each 1 μg/g increase in Ni, 10 μg/g increase in Fe, and 100 μg/g increase in Zn, the risk of total birth defects decreased by 13% (OR = 0.87, 95% CI: 0.82–0.93), 12% (OR = 0.88, 95% CI: 0.82–0.94) and 14% (OR = 0.86, 95% CI: 0.79–0.93), respectively. The concentrations of urine Al (OR = 0.90, 95% CI: 0.85–0.95), Cr (OR = 0.95, 95% CI: 0.91–0.99) and Mn (OR = 0.97, 95% CI: 0.96–0.99) were slightly negatively associated with total birth defects.

**Table 3:** Adjusted ORs and 95% CIs for the association between urinary element concentrations and birth defects.

Elements^c^	Case/control	Model 1^a^	*p*-value	Model 2^b^	*p*-value
Mg (mg/g)	271/542	0.96 (0.91–1.01)	0.115	0.97 (0.92–1.03)	0.296
Al (μg/g)	271/542	0.89 (0.85–0.94)	<0.001	0.90 (0.85–0.95)	<0.001
Ca (mg/g)	271/542	0.98 (0.95–1.01)	0.216	0.98 (0.95–1.01)	0.300
Cr (μg/g)	271/542	0.94 (0.90–0.98)	0.003	0.95 (0.91–0.99)	0.025
Mn (μg/g)	271/542	0.98 (0.96–1.00)	0.012	0.97 (0.96–0.99)	0.008
Fe (μg/g)	271/542	0.87 (0.81–0.93)	<0.001	0.88 (0.82–0.94)	<0.001
Ni (μg/g)	271/542	0.87 (0.81–0.93)	<0.001	0.87 (0.82–0.93)	<0.001
Cu (μg/g)	271/542	0.91 (0.83–1.00)	0.058	0.91 (0.83–1.01)	0.081
Zn (μg/g)	271/542	0.86 (0.80–0.94)	<0.001	0.86 (0.79–0.93)	<0.001
As (μg/g)	271/542	0.97 (0.94–1.00)	0.064	0.96 (0.93–1.00)	0.052
Se (μg/g)	271/542	0.88 (0.76–1.00)	0.056	0.90 (0.78–1.04)	0.139
Sr (μg/g)	271/542	0.99 (0.98–1.01)	0.329	1.00 (0.98–1.01)	0.484
Cd (μg/g)	271/542	0.99 (0.97–1.01)	0.322	0.99 (0.97–1.01)	0.380
I (μg/g)	271/542	1.00 (0.99–1.01)	0.769	1.00 (0.99–1.01)	0.940
Ba (μg/g)	271/542	1.00 (0.98–1.01)	0.375	1.00 (0.99–1.01)	0.671
Hg (μg/g)	271/542	0.99 (0.98–1.00)	0.039	1.00 (0.99–1.00)	0.073
Pb (μg/g)	271/542	0.99 (0.98–1.01)	0.425	1.00 (0.99–1.01)	0.554

a Model 1: unadjusted model.

b Model 2: adjusted for age, gestational age, educational level, household income, BMI, family history of birth defects, first pregnancy, conception method, prepregnancy disease status, and folic acid use.

c Mg, Ca per 10 mg/g increment, Zn per 100 μg/g increment, Al, Fe, Cu, As, Se, Sr, I, and Ba per 10 μg/g increment, Ni per 1 μg/g increment, Cr, Mn, Cd, and Pb per 0.1 μg/g increment, Hg per 0.01 μg/g increment.

### Sensitivity analysis

The results of sensitivity analysis showed that the results of Al, Cr, Mn, Fe, Ni, and Zn were basically consistent with the general population after excluding participants with a family history of birth defects, prepregnancy disease status or other conception methods. This shows that the results are relatively stable (Supplementary Table S2).

### Dose–response relationship

**Figure 2:**
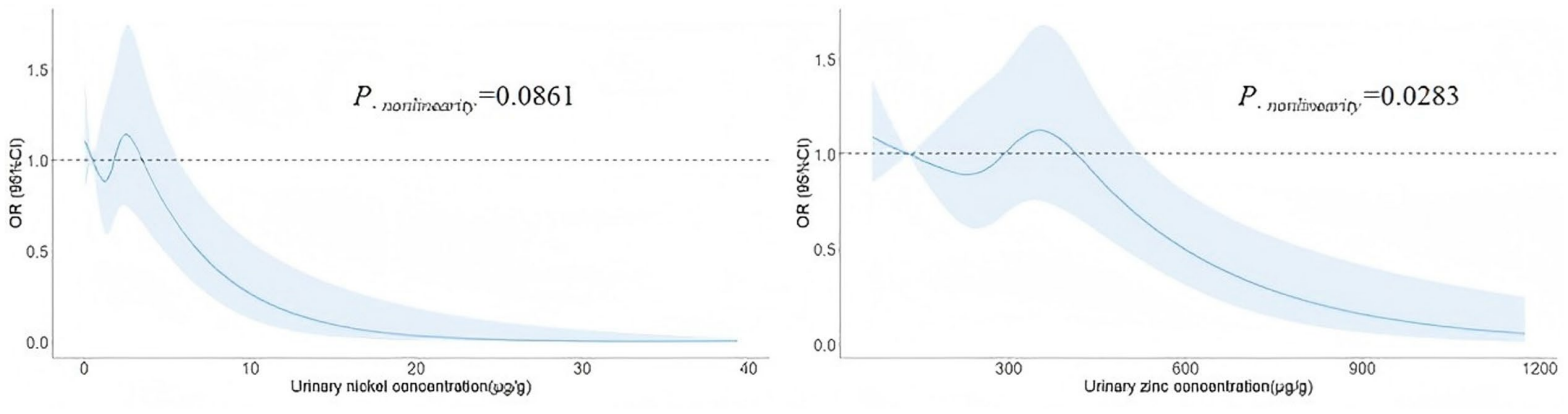
The restricted cubic spline for the association between urinary elements and birth defects. The lines represent adjusted odds ratios based on restricted cubic splines for urinary Ni and Zn in the regression model. Knots were placed at the 20th, 40th, 60th, and 80th percentiles of the urinary element distribution, and the reference value was set at the 10th percentile. Adjusted for age, gestational age, educational level, household income, BMI, family history of birth defects, first pregnancy, conception method, prepregnancy disease status, folic acid use, and statistically significant urine elements.

The results of restricted cubic spline function analysis showed that there was a significant nonlinear dose–response relationship between urinary Ni and birth defects (*P_-overall_* = 0.0015, *P_-nonlinearity_* = 0.0861), and the risk of birth defects increased with urinary Ni concentration overall. On a downward trend, there was a significant nonlinear dose–response relationship between urinary Zn and birth defects (*P*_*-overal*l_ = 0.0025, *P_-nonlinearity_* = 0.0283), and the risk of birth defects and urinary Zn concentration showed an approximately inverted “U” trend (Figure 2).

### BKMR analysis

The condPIPs of the six elements are shown in Supplementary Table S3. The condPIPs of Ni, Zn, and Fe were above 0.5, while those of the other three elements were less than 0.5. It was suggested that the coexposure of six elements to total birth defects was mainly contributed by Ni, Zn, and Fe. Ni had the highest condPIP (1.00000), which suggested that Ni had the greatest contribution to total birth defects.

**Figure 3:**
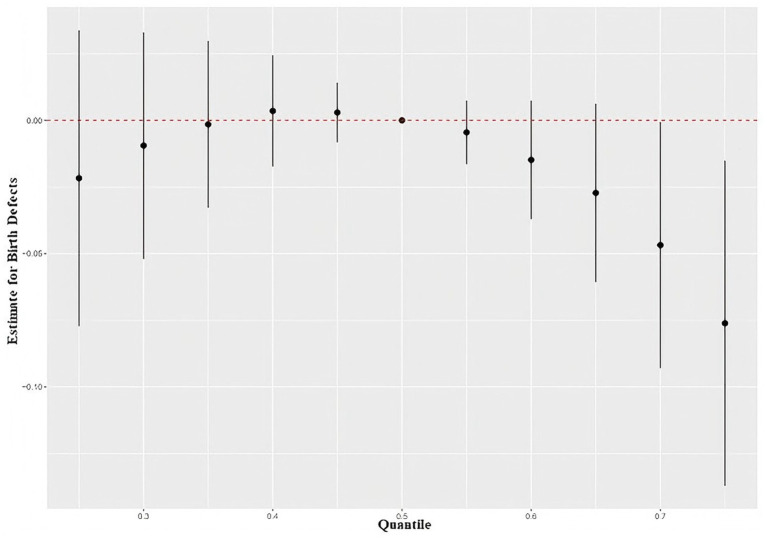
Element mixture effect on birth defects, comparing various percentiles of the mixture to the median (50th percentile).

The overall relationship between mixed element exposure and total birth defects is shown in Figure 3. Compared with the 50th percentile, the total birth defects showed a trend of first weakly rising and then declining. When the mixed exposure concentration of six elements was above the 50th percentile, the total birth defects decreased. When the mixed exposure concentration was higher than the 60th percentile, the total birth defects significantly decreased. At the low concentration of mix exposure (<25th percentile), the total birth defects slightly increased with increasing mix exposure, and this trend of increase was not significant.

**Figure 4:**
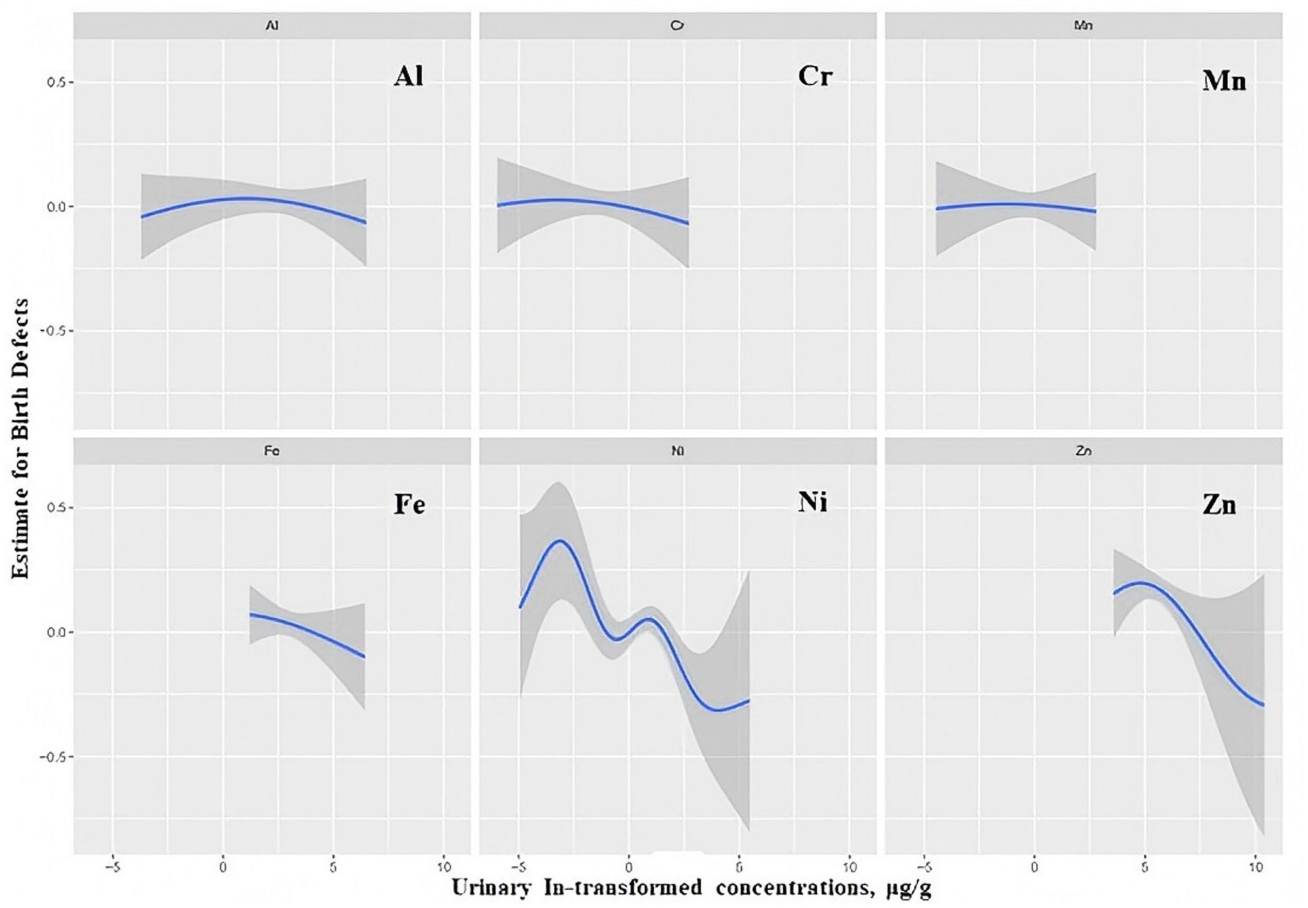
Exposure–response plots (95% CIs) for associations between log-transformed concentrations of individual elements and birth defects when all other elements were fixed at their median concentrations. Estimates were from BKMR models adjusted for age, gestational age, educational level, household income, BMI, family history of birth defects, first pregnancy, conception method, prepregnancy disease status, and folic acid use.

The change trend of the exposure-response function of the six elements is shown in Figure 4. Overall, when all the other chemicals were at their median levels, Ni and Zn showed decreasing trends associated with the total birth defects with weak increases in the lowest concentrations. Fe showed a weak negative relationship with total birth defects. It was suggested that there was a dose–response relationship between these elements and total birth defects, which was consistent with the overall effect of element mixtures on birth defects. However, Al, Cr, and Mn showed an almost flat relationship, which suggested that Al, Cr, and Mn were not significantly associated with total birth defects.

**Figure 5:**
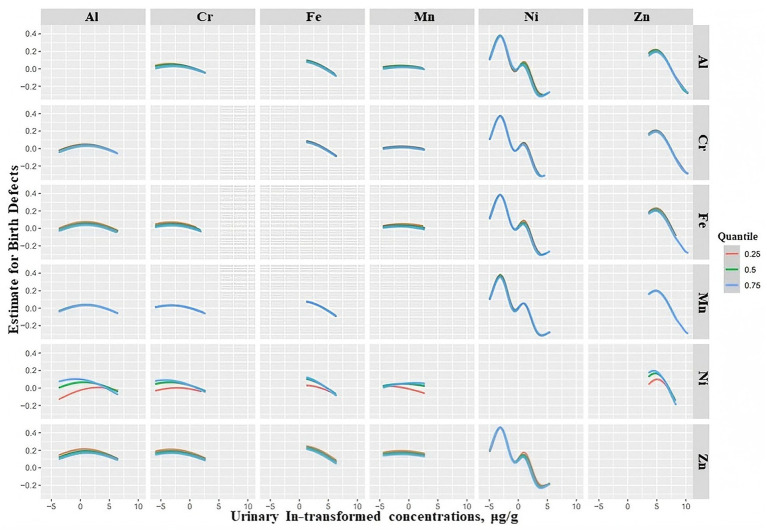
Bivariate exposure-response plots for log-transformed concentrations of individual elements and birth defects when a second metal is fixed at its 25th, 50th, or 75th percentile and the other two metals are fixed at their medians. Estimates are from BKMR models adjusted for age, gestational age, educational level, household income, BMI, family history of birth defects, first pregnancy.

The bivariate pairwise exposure–response function showed that when Al, Fe, and Mn were fixed at the median, the slope of Ni changed as the concentration of Zn increased from the 25th percentile to the 75th percentile. Similar results also existed between other elements and Ni. The results suggested that there were interactions between Ni and the other five elements (Figure 5).

## Discussion

Our study assessed the prospective association between maternal urinary multiple-element exposure concentrations in the first trimester and offspring birth defects. On the one hand, the logistic regression indicated that urinary Al, Cr, Mn, Fe, Ni, and Zn were inversely associated with total birth defects. On the other hand, considering the joint effect of elements, the BKMR results showed that birth defects showed an in apparent trend of first rising at low mix exposure (<25th percentile) and then significantly declining above the 50th percentile of mix exposure. When the element mixture was in the 70th percentile or above, it was significantly negatively correlated with birth defects.

Generally, current studies are mainly focused on the multiple adverse effects of Ni on human health, such as cancer and allergies (37, 38). Additionally, several studies have suggested that Ni is teratogenic in embryonic development using animal models (39, 40). However, there was a double face of Ni to humans and other organisms. Several studies have suggested that Ni could be beneficial for humans and animals as a “possibly essential element,” such as a positive regulatory effect on animal reproduction, growth and development, and intestinal flora (41, 42). To date, studies on the effects of Ni on birth defects have not attracted widespread attention. Several previous studies suggested that Ni exposure in the mother was not associated with adverse pregnancy outcomes or birth defects such as musculoskeletal defects and genital malformation (43–46). Liu et al. (47) found that the concentration of Ni in the umbilical cord tissue of children with NTDs was higher than that in healthy infants. Additionally, one study indicated that Ni exposure was positively associated with the risk of CHDs (48). On the other hand, two independent studies, based on 21 cases and 103 cases, respectively, both suggested that the concentrations of Ni in the maternal birth defect group were lower than those in the control group, but this difference was not significant (18, 49). Another study suggested that the concentration of Ni in maternal hair was significantly negatively associated with NTDs in offspring (50). However, one study showed that both deficiencies and excessive exposure to Ni were associated with an increased risk of NTDs using an external exposure study from soil (51). BKMR analysis suggested that the joint effect and individual effect of Ni on birth defects both slightly increased and then rapidly decreased after considering the interaction between Ni and other elements. This result was similar to a previous report, in which it was indicated that the concentration of Ni in the air and the risk of hypospadias showed an inverted U shape (52). The reason for this might be that low-dose exposure and high-dose exposure to elements have different effects on human health (53). Our analysis revealed an inverse association between Ni exposure and the risk of birth defects. Although this observation was worth noting, it must be interpreted with considerable caution. The apparent effect lacked a biological plausibility based on current evidence, and may reflect methodological limitations such as residual confounding, complex interactions with other nutritional or environmental factors, or exposure misclassification. Furthermore, the non-linear dose–response relationships typical of essential and toxic elements, or timing-specific effects during gestation, could contribute to associations. Therefore, we explicitly avoid attributing any beneficial effects to Ni solely based on the results of these epidemiological studies. Instead, we emphasized the need for rigorous mechanistic studies and replication in independent cohorts to clarify whether this association represented a causal relationship, an artifact of study design, or a context-dependent interaction. Future research should aim to characterize Ni exposure with greater temporal and chemical specificity, and to explore potential effect modifiers.

As an essential trace element, Zn participates in the composition of 2,800 kinds of proteins in the body and is of great significance to the growth and development of the human body, the immune system, and the synthesis of protein and DNA (54, 55). In our study, it was found that increased urinary Zn concentrations in the first trimester of pregnancy significantly reduced the risk of birth defects in offspring (OR = 0.86, 95% CI: 0.79–0.93). This result demonstrated that the high concentration of Zn in the urine of pregnant women was associated with a decrease in total birth defects. To date, there have been few studies on the association between urinary Zn concentrations and birth defects. However, the results of several previous studies using the concentration of Zn in serum, placenta and other tissues were consistent with this study. Several studies have found that mothers of healthy babies have higher concentrations of Zn than those with birth defects in serum and plasma (24, 56, 57). In a case–control study, compared with mothers with normal serum Zn, serum Zn-deficient mothers had more than 7 times the risk of fetal malformation (OR = 7.01, 95% CI: 2.72–18.11) (13). On the other hand, similar effects of Zn were found for multiple subtypes of birth defects. Tindula et al. (58) found that a one-unit increase in the natural logarithm of maternal toenail Zn concentrations was associated with an 89% reduction in the odds of a child developing spina bifida in the adjusted model (OR = 0.11, 95% CI: 0.03–0.42). Ni et al. found that exposure to higher concentrations of Zn in utero was associated with a reduced risk of cleft lip and palate based on a birth defect surveillance system in five rural counties in Shanxi Province, China (OR = 0.35, 95% CI: 0.14–0.86) (59). In addition, this effect of Zn against birth defects and its multiple mechanisms have been confirmed by cell and animal experiments (60). Zn deficiency in female mice led to an increased incidence of fetal cardiac malformations, which was associated with a significant reduction in placental metallothionein 1 and Zn transporter 1 mRNA expression below the threshold (61). Several previous studies suggested that a low concentration of Zn could promote the occurrence of oxidative stress and induce damage to proteins, lipids and DNA. However, oxidative stress and DNA damage are important causes of fetal development (62–65). Zn deficiency during pregnancy may also lead to abnormal folic acid metabolism, which in turn may lead to NTDs and other embryonic developmental disorders (66). However, excessive Zn exposure would also be harmful. A recent study on metal mining of parents and birth defects found that the doubling of cord blood Zn was associated with an increased risk of birth defects (OR = 5.3, 95% CI: 1.6–16.6) (67).

Our study found that increased urinary Fe concentrations in the first trimester of pregnancy were significantly associated with a decrease in total birth defects in offspring (OR = 0.88, 95% CI: 0.82–0.94). However, this negative correlation between Fe and birth defects was not significant by BKMR. One study analyzed the Fe content in the placenta, but there was no difference in the Fe concentration between the placenta of the case group and that of the control group (19). Regarding Fe, most studies have focused primarily on the preventive effect of Fe supplementation during pregnancy on birth defects. Hong et al. found that Fe supplementation, particularly during pregnancy, helped reduce the risk of cleft lip and palate (OR = 0.27, 95% CI: 0.08–0.90) (68) and that increasing Fe intake in the first month was more helpful in preventing birth defects (26). Similar results have also been reported in animal experiments. Kalisch-Smith et al. demonstrated in mice that maternal Fe deficiency increased the severity of cardiac and craniofacial defects in a mouse model (43).

According to logistic regression analysis, the urine concentrations of Al, Cr, and Mn were significantly associated with a decrease in total birth defects. However, according to the coexposure analysis, the influence of Al, Cr, and Mn on birth defects was quite limited (condPIP < 0.5). Al, Cr, and Mn were not significantly associated with total birth defects due to the flat relationship of exposure-response by BKMR, and they interacted with Ni. In previous population studies, there were few studies about the influence of Al on birth defects. A study showed that although 88 pregnant women had excess Al sulfate in their drinking water, no birth defects were found in their offspring (69). Another study found that regions with high birth defect rates were characterized by lower Al content, which seemed to be consistent with our results (70). In addition, the effects of Al on offspring animals often involve reduced fetal weight and delayed ossification rather than structural abnormalities (71). In exposure experiments involving administration of oral Al salts to female rats, Wang et al. found inhibition of reproductive function to be the more obvious effect (72). Cr is a microelement for humans. Marouani et al. (73) administered hexavalent Cr by intraperitoneal injection during organogenesis in rats and observed fetal growth retardation, facial defects, and missing tails. However, in population studies, the teratogenicity of Cr in the fetus was not clear. In their hospital-based case–control study, maternal blood Cr was not correlated with fetal CHDs (74). Another study found that there were no significant differences in the levels of Cr in amniotic fluid between the control group and case group (pregnant women with NTDs in offspring) (75). The neurotoxicity of Mn is well known (76). Most of the effects of Mn on birth defects involve mostly the neurodevelopment of the fetus but are limited only to placental tissue. It was found that the risk of fetal NTDs increased with increasing Mn concentration in the placenta (OR = 3.17, 95% CI: 2.35–4.28) (77). However, no similar association was found in other tissues, such as umbilical cord tissue and amniotic fluid (59, 75). It appeared that the effects of Mn on birth defects were limited to certain subtypes (25). In addition, both Mn deficiency and Mn excess may affect fetal development (78). Several studies have found an inverted U-shaped relationship between blood Mn concentrations and birth weight (79) or neurodevelopment (80), suggesting a dual role for Mn.

Some limitations of this study need to be addressed. First, we did not measure other maternal biological samples, such as blood, placenta, and amniotic fluid, which may make the assessment of maternal elemental exposure incomplete. Generally, urine samples, especially spot urine samples, were more convenient for sample collection than other biological samples in the assessment of elemental exposure. This advantage of urine samples could increase the compliance in participants. However, there were some shortcomings for spot urine samples in exposure assessment. The instability of the concentration of some elements in urine may bias the results of the influence of element exposure on total birth defects. This disadvantage was present in both toxic elements and trace elements. The intraclass correlation coefficient (ICC) was an important indicator for the stability of the concentration of elements in urine. Usually, elements with fast turnover rates have low ICCs. The ICCs of some elements (such as As, Cd, Pb, Ni, and Mn) in spot urine were above 0.5, which suggested that the concentrations of these elements were at least fair to good reliability. The ICCs of Zn and Al in spot urine were 0.37 and 0.39, respectively, which suggested that the concentrations of Zn and Al had poor reliability. Additionally, the concordance coefficients of Zn and Al between spot urine and 24-h urine were both higher than 0.5, which indicated that there was moderate agreement between spot and 24-h metal concentrations for Zn and Al (81). While urinary measurement was well-validated and commonly used for biomonitoring of many elements with relatively short biological half-lives, it may not be the optimal biomarker for all elements studied. For certain metals, urinary excretion primarily reflected recent exposure or circulating levels rather than the long-term body burden accumulated in tissues. This could lead to potential misclassification of the true long-term exposure status. Our study attempted to mitigate this by standardizing creatinine and adjusting for specific gravity. Nonetheless, we cannot rule out the possibility that for some elements, urinary levels may either overestimate or underestimate the biologically relevant dose at the target tissue. Future studies incorporating multiple matrices or repeated sampling would provide a more robust assessment of exposure profiles. Therefore, with comprehensive consideration of reliability and convenience, spot urine samples could be used to investigate the relationship between multiple element exposure and birth defects despite the insufficient reliability of some elements (82). Second, the element concentrations observed in our study cannot be contextualized with environmental exposure background, but the element concentrations in our study population fall within the range reported in previous studies for the general population in China (83). This suggested that our findings likely reflect variations at environmental background levels rather than extreme deficiency or overt toxicity. However, the absence of universally established ‘normal’ or “deficient” ranges for many elements in maternal biological matrices posed a significant challenge. It remained difficult to definitively ascertain whether the observed associations were driven by a response to suboptimal nutritional status, low-level environmental exposure, or a complex interplay of both. Future studies would benefit from concurrently assessing dietary intake, environmental sources, and established clinical biomarkers to better contextualize elemental concentrations and distinguish between deficiency-driven and exposure-driven effects. Third, we have attempted an exploratory analysis of different subtypes of birth defects, in which zinc still showed significance for craniofacial abnormalities and genitourinary abnormalities. Due to the small number of cases in individual subtypes, it was not possible to conduct a statistically reliable and formal subtype-specific analysis (Supplementary Table S4). However, in this study, the number of cases with multiple congenital diseases at the same time exceeded 20%. The association between element exposure and total birth defects was more concerning due to the limited number of cases. In addition, regarding the basic characteristics of the study population, due to the exclusion of some participants who lacked urine samples, there was a certain bias in the representativeness of the study population included in the analysis. However, we also conducted an additional analysis of the basic characteristics of the excluded study population, and the results were not significantly different from the population included in the analysis. Therefore, the current population still had good representativeness (Supplementary Table S5). Furthermore, although a 1:1 ratio was typically the initial design choice, increasing the number of controls per case can enhance the statistical power of the analysis, especially when the number of available cases was limited. Although a 1:4 study design yielded more significant results, considering the higher difficulty and cost of epidemiological investigations when the study subjects were pregnant women, and taking into account the matching criteria (maternal age and gestational age), a 1:2 ratio was a traditional and widely accepted compromise, which aimed to ensure comparability between groups while maintaining a feasible pool of eligible controls from the source population. However, this study still had several advantages. This was a study to assess the association between multiple-element exposures and total birth defects in the first trimester based on a Chinese population. All surveys and sample collections were conducted in the first trimester, which minimized recall bias and ensured that the samples accurately represented exposure to maternal elements during fetal organ development.

## Conclusion

The current results show that the above 50th percentile concentration of a mixture of Al, Cr, Mn, Fe, Ni, and Zn in urine during the first trimester of pregnancy was significantly negatively associated with total birth defects. In the future, the results of this study need to be further replicated, and the underlying mechanisms should be explored to better elucidate the effects of maternal element exposure on birth defects.

## Data Availability

The original contributions presented in the study are included in the article/Supplementary material, further inquiries can be directed to the corresponding authors.
