# Supplementary material for: Multiple-element exposure in early pregnancy and birth defects: a nested case–control study based on the China birth cohort study
Source: Front Nutr. 2026 Mar 9;13:1722672. doi: 10.3389/fnut.2026.1722672 (PMC13006698; doi:10.3389/fnut.2026.1722672)
Supplement: Supplementary file 1 [file Supplementary_file_1.docx]

Supplementary Material

Table S1. Limits of detection, percentages of samples below detection limits.

Table S2. Sensitivity analysis of elements and birth defects.

Table S3. The condPIP of the six elements.

Table S4.Adjusted ORs and 95% CIs for the association between urinary element concentrations and birth defects subtypes.

Table S5. Basic characteristics of people with or without urine samples

Figure S1. Correlations between urinary elements among the case-control participants.

**Table S1.** Limits of detection, percentages of samples below detection limits.

| Elements | LOD (ng/mL) | Total population < LOD^a^(%) | Cases < LOD^a^(%) | Controls < LOD^a^(%) |
| --- | --- | --- | --- | --- |
| Mg | 0.115 | 4(0.5) | 0 | 4(0.7) |
| Al | 0.120 | 53(6.5) | 8(3.0) | 45(8.3) |
| Ca | 0.458 | 0 | 0 | 0 |
| Cr | 0.007 | 14(1.7) | 0 | 0 |
| Mn | 0.025 | 3(0.4) | 0 | 3(0.6) |
| Fe | 0.340 | 0 | 0 | 0 |
| Ni | 0.021 | 16(2.0) | 9(3.3) | 7(1.3) |
| Cu | 0.022 | 0 | 0 | 0 |
| Zn | 0.105 | 0 | 0 | 0 |
| As | 0.034 | 0 | 0 | 0 |
| Se | 0.044 | 0 | 0 | 0 |
| Sr | 0.059 | 0 | 0 | 0 |
| Cd | 0.008 | 1(0.1) | 0 | 1(0.2) |
| I | 0.042 | 1(0.1) | 0 | 1(0.2) |
| Ba | 0.005 | 0 | 0 | 0 |
| Hg | 0.039 | 174(21.4) | 53(19.6) | 121(22.3) |
| Pb | 0.009 | 0 | 0 | 0 |

Abbreviations: LOD, limit of detection;

^a^ Number and percentage of samples below LOD.

**Table S2.** Sensitivity analysis of elements and birth defects.

| Case/Control | Model 1^b^ | *P value* | Model 2^c^ | *P value* |
| --- | --- | --- | --- | --- |
| **Family history of birth defects** |  |  |  |  |
| **No (259/520)** |  |  |  |  |
| **Al** | 0.89(0.84-0.94) | ＜0.001 | 0.89(0.85-0.94) | ＜0.001 |
| **Cr** | 0.94(0.91-0.99) | 0.007 | 0.95(0.91-0.99) | 0.014 |
| **Mn** | 0.98(0.96-0.99) | 0.022 | 0.98(0.96-0.99) | 0.018 |
| **Fe** | 0.88(0.82-0.93) | ＜0.001 | 0.88(0.83-0.94) | ＜0.001 |
| **Ni** | 0.88(0.83-0.93) | ＜0.001 | 0.88(0.83-0.93) | ＜0.001 |
| **Zn** | 0.86(0.80-0.94) | ＜0.001 | 0.86(0.79-0.94) | ＜0.001 |
| **Conception method** |  |  |  |  |
| **Natural conception (243/521)** |  |  |  |  |
| Al | 0.89(0.84-0.94) | ＜0.001 | 0.89(0.84-0.94) | ＜0.001 |
| Cr | 0.94(0.90-0.98) | 0.005 | 0.94(0.90-0.98) | 0.005 |
| Mn | 0.97(0.96-0.99) | 0.009 | 0.97(0.95-0.99) | 0.006 |
| Fe | 0.86(0.80-0.92) | ＜0.001 | 0.86(0.80-0.92) | ＜0.001 |
| Ni | 0.86(0.80-0.92) | ＜0.001 | 0.86(0.80-0.92) | ＜0.001 |
| Zn | 0.85(0.77-0.92) | ＜0.001 | 0.84(0.77-0.92) | ＜0.001 |
| **Pre-pregnancy disease status** |  |  |  |  |
| **No (202/445)** |  |  |  |  |
| Al | 0.90(0.85-0.95) | ＜0.001 | 0.90(0.85-0.95) | ＜0.001 |
| Cr | 0.95(0.90-0.99) | 0.021 | 0.95(0.91-0.99) | 0.033 |
| Mn | 0.98(0.96-1.00) | 0.081 | 0.98(0.96-1.00) | 0.090 |
| Fe | 0.86(0.80-0.93) | ＜0.001 | 0.87(0.80-0.93) | ＜0.001 |
| Ni | 0.87(0.81-0.93) | ＜0.001 | 0.87(0.81-0.93) | ＜0.001 |
| Zn | 0.85(0.78-0.93) | 0.001 | 0.84(0.76-0.93) | ＜0.001 |

^a.^ Unconditional logistic regression model was used for sensitivity analysis.

^b.^ Model 1: Unadjusted model.

^c.^ Model 2: Adjusted for age, gestational weeks, education level, household income, BMI, smoking status, drinking status, family history of birth defects, first pregnancy, conception method, pre-pregnancy disease status, and folic acid use.

**Table S3.**The condPIP of the six elements.

| Elements | CondPIP |
| --- | --- |
| AI | 0.40656 |
| Cr | 0.25672 |
| Mn | 0.30456 |
| Fe | 0.56376 |
| Ni | 1.00000 |
| Zn | 0.83408 |

**Table S4.**Adjusted ORs and 95% CIs for the association between urinary element concentrations and birth defects subtypes.

|  |  | Craniofacial abnormalities(n=49) | Limb abnormalities(n=44) | Digestive system abnormalities(n=13) | Congenital heart disease(n=61) | Chromosomal abnormalities(n=53) | Urogenital system abnormalities(n=41) | Others(n=10) |
| --- | --- | --- | --- | --- | --- | --- | --- | --- |
| AI | OR(95%CI) | 0.67(0.50-0.91) | 0.86(0.71-1.04) | 1.01(0.45-2.30) | 0.88(0.75-1.03) | 0.97(0.87-1.09) | 0.95(0.87-1.04) | 0.56(0.18-1.73) |
|  | *P* | 0.010 | 0.129 | 0.975 | 0.102 | 0.631 | 0.306 | 0.313 |
| Cr | OR(95%CI) | 0.96(0.87-1.05) | 0.77(0.63-0.93) | 1.02(0.33-3.20) | 0.98(0.88-1.09) | 0.99(0.90-1.11) | 1.02(0.91-1.14) | 0.52(0.17-1.57) |
|  | *P* | 0.391 | 0.008 | 0.971 | 0.654 | 0.958 | 0.791 | 0.247 |
| Mn | OR(95%CI) | 0.96(0.91-1.02) | 0.96(0.90-1.01) | 0.77(0.19-3.05) | 0.99(0.95-1.03) | 1.01(0.96-1.06) | 0.95(0.89-1.01) | 0.68(0.22-2.10) |
|  | *P* | 0.193 | 0.109 | 0.708 | 0.668 | 0.738 | 0.115 | 0.504 |
| Fe | OR(95%CI) | 0.81(0.67-0.99) | 0.78(0.60-1.01) | 1.01(0.14-7.37) | 0.88(0.74-1.04) | 1.01(0.88-1.16) | 0.82(0.67-1.00) | 0.43(0.10-1.93) |
|  | *P* | 0.035 | 0.055 | 0.994 | 0.130 | 0.865 | 0.050 | 0.272 |
| Ni | OR(95%CI) | 0.80(0.63-1.01) | 0.82(0.64-1.04) | 0.99(0.77-1.27) | 0.94(0.85-1.03) | 0.90(0.77-1.06) | 0.86(0.69-1.07) | 0.94(0.77-1.15) |
|  | *P* | 0.063 | 0.106 | 0.923 | 0.178 | 0.201 | 0.183 | 0.572 |
| Zn | OR(95%CI) | 0.97(0.94-0.99) | 0.98(0.95-1.01) | 1.03(0.71-1.50) | 0.99(0.97-1.01) | 0.99(0.97-1.01) | 0.97(0.94-0.99) | 0.96(0.81-1.13) |
|  | *P* | 0.029 | 0.063 | 0.876 | 0.226 | 0.455 | 0.018 | 0.612 |

Note:Adjusted for age, gestational weeks, education level, household income, BMI, smoking status, drinking status, family history of birth defects, first pregnancy, conception method, pre-pregnancy disease status, and folic acid use.

**Table S5.**Basic characteristics of people with or without urine samples

|  | Yes (n=271) | No (n=137) | *P value ^a^* |
| --- | --- | --- | --- |
| Age (years) | 30.91±4.17 | 31.97±4.60 | 0.020 |
| Gestational age (weeks) | 10.30±1.96 | 10.33±1.95 | 0.871 |
| Educational level, n (%) |  |  | 0.669 |
| Junior high school and below | 11(4.1) | 5(3.6) |  |
| High school | 40(14.8) | 16(11.7) |  |
| University and above | 220(81.1) | 116(84.7) |  |
| Household income, n (%) |  |  | 0.561 |
| Below 50,000 CNY^b^ | 13(4.8) | 6(4.4) |  |
| 50,000-200,000 CNY^b^ | 96(35.4) | 56(40.9) |  |
| Above 200,000 CNY^b^ | 162(59.8) | 75(54.7) |  |
| Smoking status, n (%) |  |  | 0.865 |
| Smoking | 267(98.5) | 136(99.3) |  |
| Never smoking | 4(1.5) | 1(0.7) |  |
| Drinking status, n (%) |  |  | 0.300 |
| Drinking | 7(2.6) | 7(5.1) |  |
| No drinking | 264(97.4) | 130(94.9) |  |
| BMI | 21.32±2.99 | 21.16±3.07 | 0.610 |
| First pregnancy, n (%) |  |  | 0.607 |
| Yes | 102(37.6) | 48(35.0) |  |
| No | 169(62.4) | 89(65.0) |  |
| Conception method, n (%) |  |  | 0.789 |
| Natural conception | 243(89.7) | 124(90.5) |  |
| Others | 28(10.3) | 13(9.5) |  |
| Prepregnancy disease status^c^, n (%) |  |  | 0.642 |
| Yes | 69(25.5) | 32(23.4) |  |
| No | 202(74.5) | 105(76.6) |  |
| Family history of birth defects, n (%) |  |  | 0.205 |
| Yes | 12(4.4) | 2(1.5) |  |
| No | 259(95.6) | 135(98.5) |  |
| Folic acid use, n (%) |  |  | 0.007 |
| Using | 225(83.0) | 98(71.5) |  |
| No using | 46(17.0) | 39(28.5) |  |

1. *P*_-_values were derived from Student’s t-tests for continuous variables according to the data distribution and the chi-square test for the categorical variables.
2. CNY was Chinese yuan.
3. Prepregnancy disease including diabetes, hypertension, periodontal disease, and reproductive tract inflammation in the first three months of pregnancy.


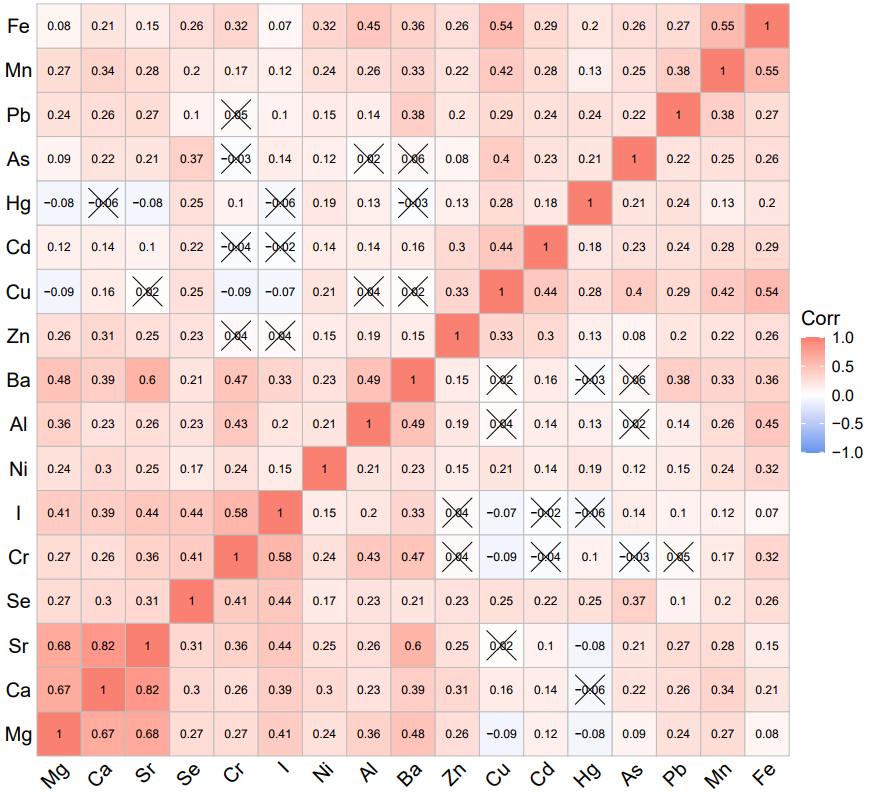


**Figure S1**. Correlations between urinary elements among the case-control participants.

“×” means not significant.
